# Situational factors affecting abstinence from drugs: Panel data analysis of patients with drug use disorders in residential drug use treatment

**DOI:** 10.1002/pcn5.174

**Published:** 2024-02-20

**Authors:** Satomi Mizuno, Takuya Shimane, Satoshi Inoura, Toshihiko Matsumoto

**Affiliations:** ^1^ Department of Drug Dependence Research National Center of Neurology and Psychiatry, National Institute of Mental Health Tokyo Japan; ^2^ Department of Forensic Medicine, Graduate School of Medicine The University of Tokyo Tokyo Japan

**Keywords:** abstinence, alcohol drinking, drug rehabilitation center, drug use disorder, follow‐up study

## Abstract

**Aim:**

To identify situational factors that can predict drug abstention in patients with drug use disorders undergoing residential drug use treatment.

**Methods:**

Patients with drug use disorders admitted to drug addiction rehabilitation centers (DARCs) in 2016 were involved in this study. Longitudinal panel data were used, with eight follow‐up surveys over 6 years, approximately every 6 months. Of the 2752 samples from the eight follow‐up surveys, 2293 were analyzed as the complete panel data set. The primary outcome was drug abstention for approximately 6 months. The influences of situational factors during this period on the primary outcome were also assessed using a generalized linear mixed model in which inter‐individual differences were controlled as variable effects.

**Results:**

The use of residential DARCs positively influenced the primary outcome (adjusted odds ratio [AOR] 3.33, 95% confidence interval [CI] 1.79–6.21) when compared to no DARC usage. The cessation of drinking also positively affected the primary outcome (AOR 3.10, 95% CI 1.79–4.62), while employment status (AOR 2.22, 95% CI 1.12–4.41) and the cessation of drinking (AOR 4.92, 95% CI 2.77–8.72) positively impacted the primary outcomes of patients not using DARCs.

**Conclusion:**

The use of residential DARCs and the cessation of drinking positively affected drug abstention rates. Employment and the cessation of drinking for patients who were not using the DARCs also had a positive effect. This information will aid in the development of social recovery strategies for people with drug use disorders.

## Graphical abstract

This study involved people with drug use disorders who participated in a 12‐step program managed by recovered drug users in the rehabilitation facility group. We assessed the associations between abstinence from drugs and the status of patients' facility use, living location, employment, receiving welfare, and sobriety. We found that the state of being discharged from facilities negatively influenced abstinence from drugs whereas sobriety positively influenced abstinence.

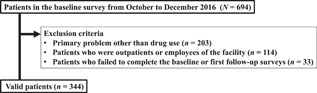

## INTRODUCTION

The treatment of persons with drug use disorders has become increasingly important worldwide because of the association with physical and mental health disorders, economic instability, and the loss of a healthy social life.^1^ An estimated 31 million people worldwide have lost healthy social lives as a result of disabilities and premature deaths caused by drug use disorders.^2^ The 2030 Agenda for Sustainable Development and Sustainable Development Goals (SDGs) aim to “strengthen the prevention and treatment of drug abuse.”^3^ Drug abstinence after treatment programs is of significant interest to patients with drug use disorders living in some countries, including Japan, where drug consumption is recognized as criminal and this is strictly enforced. Information regarding the situations that enable continued abstinence after treatment programs could be informative for people with drug use disorders who are trying to reconcile continued abstinence with independent social lives.

Residential addiction treatment, such as with drug addiction rehabilitation centers (DARCs), is a well‐known rehabilitation model for drug addiction and a therapeutic community model. Many previous studies have shown that patient populations who have completed residential addiction treatments have reduced drug use when compared with those who have not.^4^, ^5^, ^6^, ^7^ Furthermore, the patient population experiencing drug use relapse had the following characteristics: comorbid psychiatric disorders,^5^, ^8^ criminal record,^7^ divorce,^7^ family history of addiction,^7^ severity of the problem with drugs,^7^ living location such as their houses,^7^ unemployment,^7^ being a student,^7^ and poor aftercare participation after completing residential treatment.^7^, ^9^

Previous studies^4^, ^5^, ^6^, ^7^ have identified factors influencing abstinence status after residential addiction treatment programs by examining the baseline characteristics of the patient population who experienced drug relapse when compared with the patient population who did not. However, it is also important to examine the relationship between the changes in the patient's situation and the changes in abstinence status at an intra‐individual level while controlling for individual differences and time‐varying effects, such as those at the follow‐up time points, as well as the relationship between the characteristics of the patient population at baseline and their abstinence status at the end of the follow‐up. This is because each patient's situation is likely to change over time, for example unemployed people may become employed or people living in institutions may move houses, and their baseline circumstances will thus not remain the same throughout the follow‐up period. Furthermore, previous studies have reported patient characteristics and situational factors influencing abstinence by comparing information at the baseline in the nonrelapsed patient population with that in the relapsed patient population. However, it is unclear whether the results of these studies indicate the characteristics of the nonrelapsed patient population associated with abstinence status or whether these show the individual‐level situation changes associated with or a mixture of the two. Owing to the complex patterns of stability and changes in drug use in each patient, studies should be designed to predict changes in abstinence status at the intra‐individual level, while controlling for inter‐individual differences to assess the impacts of the patient's situation.^10^, ^11^ A panel survey in which the same question or test is repeated several times for the same person is an effective method to assess the degree of changes in situation X (e.g., employment, unemployment, admission, discharge, living at home, living in an institution) that occurs at the intra‐individual level to predict the dependent variable Y (e.g., continued abstinence or not) that occurs at the intra‐individual level while controlling for inter‐individual differences (e.g., sex, age, and medical history). In particular, a panel data analysis avoids the missing variable bias because the effects of the unknown personal factors on the independent variables can be controlled for.

A panel data analysis of patients with drug use disorders in DARCs, controlling for inter‐individual differences, was used in this study to assess the extent to which changing circumstances at the individual level (e.g., the situation of patients' facility usage, living location, employment situation, situation of receiving welfare, and situation of no drinking) can predict changes in abstinence status.

## METHODS

### Data sources and ethical considerations

This study used secondary data from the pubication “Follow‐up study of DARC users in Japan,”^12^ a self‐reported study. DARCs are rehabilitation facilities that follow a 12‐step program and are predominantly established and run by staff who have experienced problems with drug use and recovered from drug dependence by attending meetings based on the program. DARCs are intermediate facilities that provide a place to live for social recovery.^13^, ^14^ Staff and facility users seek to facilitate honest confessions from facility users who have consumed drugs and alcohol; confessions are not forced, and no punishment is implemented. The ethics committees of our institution approved this study (A2016–022), which conformed to the provisions of the Declaration of Helsinki.

### Patients and procedures in the original study

The original study design was a prospective cohort study that included eight follow‐up surveys over 6 years, from October 2016 to November 2022. The original study obtained a panel data set in which the same questions were repeatedly asked to the same patient at each follow‐up, using self‐administered questionnaires and interviews. Referring to a previous study that tracked abstinence status,^9^ each patient was followed up every approximately 6 months to investigate their abstinence status. Relapse was ascertained through self‐reporting during each survey. The first four follow‐ups (October 2016 to October 2018) were conducted every 6 months. The subsequent four follow‐up surveys (June 2019 to October 2021) were conducted every 8 months because of the coronavirus disease 2019 pandemic. The details of the survey procedure are described in the original study.^12^, ^15^ All facilities nationwide were contacted to obtain consent from facility directors and staff to cooperate with this study. Next, the patients received an explanation of the purpose of the study from the staff, and interested patients could participate in the study after providing written informed consent. The patients were asked to complete a self‐administered questionnaire as a baseline survey to collect their background data. The eight follow‐up surveys were conducted face‐to‐face or via telephone interviews with DARC staff depending on the patient's facility use at the time of the follow‐up: face‐to‐face interviews for inpatients and outpatients, and telephone interviews mainly for discharged patients. Self‐administered questionnaires were completed, and then informed consent was obtained from 694 of 701 patients from 46 of the 57 DARC facilities nationwide. As the recruitment of study participants was outsourced to the heads of the centers and the patients were asked to complete a self‐administered questionnaire and an interview under an anonymized name that each patient determined themselves, the data provided by the centers did not contain personal details of the patients.

### Patient and sample selection

**Figure 1:**
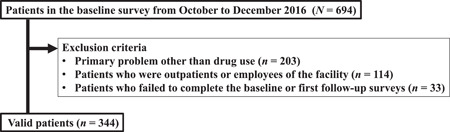
Flowchart of patient selection.

From the 694 patients in the original study, the data for 344 patients were extracted. All of these patients (1) were facility users who were residents of the facility and participated in the daytime treatment program as residential patients, (2) had drug use disorders as the cause of their primary addiction, and (3) completed the baseline and the first and second follow‐up surveys. Outpatients and staff were excluded to maintain the homogeneity of the target population at the baseline. A flowchart of the patient selection process is shown in Figure 1. The proportions of responses for the first, second, third, fourth, fifth, sixth, seventh, and eighth follow‐up surveys against the baseline were 100.0%, 100.0%, 93.0%, 89.2%, 84.6%, 71.8%, 64.5%, and 63.4%, respectively. Compared with the 218 patients who could participate in all follow‐up interviews, the 126 patients who could not participate did not show any differences in other characteristics in the baseline survey, apart from having a high proportion of men (P < 0.05). The proportion of patients using residential DARCs was 100.0%, 82.3%, 67.7%, 58.7%, 50.8%, 42.4%, 31.9%, and 28.5% in the first, second, third, fourth, fifth, sixth, seventh, and eighth follow‐up surveys, respectively.

### Variables

The background characteristics of the patient population were determined using self‐administered baseline survey data. To characterize the target population, we obtained information on age, sex, education (less than or completed high school), mental disorders other than substance use disorders, dependence severity, types of drugs mainly used in the past, drug‐related criminal history, and length of participation in residential addiction treatment programs. The severity of problems with drugs was evaluated using the Japanese version of the Drug Abuse Screening Test‐20 (DAST‐20).^16^ The DAST‐20 defines the severity of drug dependence as nonsevere and low (scores of 0–5), intermediate (scores of 6–10), and severe (scores ≥11). We also recorded participation in rehabilitation programs (favorable or not), relationships with other facility users and staff (favorable or not), sponsor presence, and length of stay at the facility. These variables were chosen based on a previous report.^4^, ^7^

For the panel data analysis, the dependent variable was defined as the situation in which a patient did not use drugs in the period between each follow‐up, which was a binary variable. If a patient did not use drugs between follow‐ups (approximately 6 months), the situation was classified as “no drug use.” Conversely, if the patient used drugs at least once between follow‐ups, the situation of the patient was classified as “using drugs.”

The independent variables were defined as variables of the situation, such as patients' facility usage, living location, employment, welfare receipt, and cessation of drinking. These situational variables were chosen based on the findings of previous studies. They were defined as the patient's situation at each term between the last follow‐up and the time before one previous follow‐up. Patients' facility usage was classified into three situations: not using DARCs, using residential DARCs, and using outpatient DARCs, which were dummy variables. Not using DARCs was defined as a situation in which the patient was not a DARC resident and did not participate in the outpatient treatment program. Using residential DARCs was defined as a situation where a patient lived in DARCs and participated in the treatment program. Using outpatient DARCs was defined as a situation in which an outpatient was not a DARC resident and participated only in the outpatient daytime treatment program. Living location referred to where the patient lives and sleeps. There were three categories: living at the DARC, living at home, and living at another facility apart from the DARC, which were dummy variables. If a situation involving employment occurred between follow‐ups, it was defined as an “employed situation.” If a situation involved receiving welfare between follow‐ups, it was defined as “the situation of receiving welfare.” As alcohol consumption is reportedly affected by the presence or absence of regulatory skills and habits, as well as by drug use, it was investigated as an independent variable in this study.^17^ If a patient did not drink alcohol between each follow‐up, the situation was defined as “no drinking.”

### Statistical analysis

**Figure 2:**
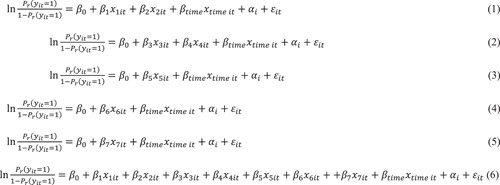
Models used in the univariable and multivariable analyses of all data sets. Coefficients indicate the following: *x*_1_, the use of residential DARC; *x*_2_, the use of outpatient DARC; *x*_3_, living location at home; x_4_, living location at facilities other than DARCs; *x*_5_, employment situation; *x*_6_, receipt of welfare; x_7_, nondrinking status; *x*_time_, follow‐up time. Each independent variable and time point variable was included in the univariate models (1–5), and all independent and follow‐up time variables were included in the multivariate model (6). The reference for situations involving residential and outpatient DARCs was based on situations in which DARC was not used. The reference for living locations at home and other facilities apart from the DARCs was based on the living location at the DARC. DARC, drug addiction rehabilitation center.

**Figure 3:**
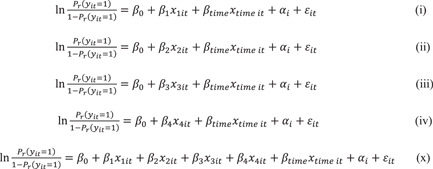
Models were used in the univariable and multivariable analyses of the nonusing drug addiction rehabilitation center (DARC). Coefficients indicate the following: *x*_1_, living location at home; x_2_, receipt of welfare; x_3_, employment status; *x*_4_, non‐drinking status; *x*_time_, follow‐up time. Each independent variable and time‐point variable were included in the univariate models (i–iv). All independent and follow‐up time variables were controlled in the multivariate model (x). The reference for living location at home was based on the living locations at facilities other than DARCs.

The baseline characteristics of the study population were described using descriptive statistics. Generalized linear mixed models were then utilized with a random intercept, using R software version 4.2.2 (R Foundation for Statistical Computing) to quantify the impact of each independent variable on the dependent variable. Of the 2752 samples from 344 patients at the eight follow‐up surveys, 2293 had responses to all questions about situational factors and abstinence and were included in the analysis. Each independent variable and the follow‐up time variable in the univariable models (Figure 2, models (1)–(5)) were controlled for, as were all independent and follow‐up time variables in the multivariable model (Figure 2, model (6)). Additionally, we also controlled for sex as an independent variable in the univariate model, as sex differences were associated with abstinence after residential addictive treatment.^4^ However, the sex‐independent variable was not significant in the models. The results suggested that the effect of the sex variable is controlled in the random effect attributed to individuals when analyzed using generalized linear mixed models. Thus, we did not include sex in the multivariate model (Table S1). In addition to the primary analysis, 634 out of 2293 samples in the univariable models (Figure 3, models (i)–(iv)) and multivariable model (Figure 3, model (x)) were analyzed in the stratified analysis of the situation when DARCs are not used.

## RESULTS

### Patient population characteristics

The background characteristics of patients are presented in Table 1. The mean age was 40.8 (standard deviation [SD] 9.9) years, patients were predominantly males, approximately half had not completed high school, one‐third had mental disorders, and approximately half had methamphetamine use disorder. Furthermore, the overall severity of the problem with drugs was classified as severe (mean DAST‐20 score 13.3, SD 4.0) and approximately half of the patients had a drug‐related criminal history. The proportions of patients classified as employed and welfare recipients were 17.2% and 79.1%, respectively. Most patients participated positively in the rehabilitation programs and had favorable relationships with users, staff, and sponsors (82.2%, 90.7%, 92.2%, and 77.9%, respectively). The median length of stay in the facilities was 20.0 months (interquartile range [IQR] 18.8–21.2).

**Table 1:** Characteristics of the patient population in the baseline survey.

	*N* = 344	Missing value (%)
Age (years), mean ± SD	40.8 ± 9.9	0
Biological sex		0
Male	319 (92.7)	
Female	25 (7.3)	
Not completed high school	188 (54.7)	0
Diagnosis of mental disorder other than addictions	133 (39.0)	10.8
Types of drugs that were used mainly in the past		0
Methamphetamine	204 (59.3)	
New psychoactive substances	51 (14.8)	
Organic solvent	25 (7.3)	
Prescription drug overdose	20 (5.8)	
Over‐the‐counter drug overdose	16 (4.7)	
Marijuana	12 (3.5)	
Others	16 (4.7)	
Severity: DAST‐20 score, mean ± SD	13.3 ± 4.0	
Drug‐related criminal history	148 (43.0)	0
Employment	59 (17.2)	0
Welfare recipient	272 (79.1)	0
Cessation of drinking	306 (89.0)	0
Abstinence from drugs^a^	326 (94.7)	0
Positive participation in rehabilitation programs	285 (82.2)	0
Favorable relationships with other facility users	312 (90.7)	0
Favorable relationships with staff	317 (92.2)	0
Presence of sponsors	268 (77.9)	0
Length of stay (months), median [IQR]	20.0 (18.8–21.2)	

*Note*: Data are presented as *n* (%) unless otherwise indicated. The other drugs included cocaine, heroin, MDMA, and gas.

Abbreviations: DAST‐20, Drug Abuse Screening Test 20; IQR, interquartile range; MDMA, 3, 4‐methylenedioxymethamphetamine; SD, standard deviation.

^a^ This variable was drawn from the first follow‐up survey. Variables without asterisks were drawn from the baseline survey.

### Factors associated with drug abstinence

The results of the univariate and multivariate analyses are summarized in Tables S1 and 2, respectively. The multivariable analysis showed that the situation when a patient is using DARCs was a significant indicator that they would have continued abstinence from drugs (adjusted odds ratio [AOR] 3.33, 95% confidence interval [CI] 1.79–6.22) when compared with the situation when a patient is not using DARCs. Furthermore, the cessation of drinking alcohol also significantly predicted continued abstinence from drugs (AOR 3.11, 95% CI 2.09–4.62).

**Table 2:** Multivariable analysis results.

Characteristic	AOR (95% CI)	P value
Situation of patients' facility usage		
Not using DARC	Reference	ー
Using residential DARC	3.33 (1.79–6.22)	<0.001
Using outpatient DARC	1.82 (0.78–4.25)	0.166
Living location		
DARC	Reference	ー
Home	0.85 (0.41–1.73)	0.647
Other facilities apart from DARCs	1.08 (0.59–1.98)	0.801
Employed situation	1.13 (0.77–1.65)	0.523
Receiving welfare	1.12 (0.71–1.74)	0.620
No drinking	3.11 (2.09–4.62)	<0.001

*Note*: The reference for the situation when a resident or outpatient was using DARC was based on not using DARC. The reference for living locations at home and other facilities apart from the DARCs was based on the living location at the DARC. The analysis was adjusted for variables at the follow‐up time. Model (6) in Figure 2 was used for this analysis.

Abbreviations: AOR, adjusted odds ratio; CI, confidence interval; DARC, drug addiction rehabilitation center.

### Stratified analysis of the situation when DARCs are not used

Univariate and multivariate analysis results for patients who did not use DARCs are presented in Tables S2 and 3, respectively. Employment (AOR 2.22, 95% CI 1.12–4.41) and the cessation of drinking (AOR 4.92, 95% CI 2.77–8.72) were significant predictors that a patient would continue to abstain from drug use.

**Table 3:** Multivariable analysis results for the stratified analysis of not using DARCs.

	AOR (95% CI)	P value
Living location: home	0.73 (0.37–1.44)	0.368
Employed situation	2.22 (1.12–4.41)	0.023
Receiving welfare	1.07 (0.56–2.08)	0.830
No drinking	4.92 (2.77–8.72)	<0.001

*Note*: The analysis was adjusted for variables at the follow‐up time. The reference for living location at home was based on living locations at facilities other than the DARCs. Model (*x*) in Figure 3 was used for this analysis.

Abbreviations: AOR, adjusted odds ratio; CI, confidence interval; DARC, drug addiction rehabilitation center.

## DISCUSSION

A panel data analysis was conducted for patients with drug use disorders in DARCs to assess how changes in individual‐level situations, such as institutional use status, place of residence, employment status, receipt of welfare, and nondrinking status, would affect their abstinence status at the individual level. The use of DARCs and the cessation of drinking were found to be significant predictors of drug abstinence. In addition to the cessation of drinking, employment was also identified as a significant predictor of drug abstinence for patients who were not using DARCs.

As many previous studies have reported on the effectiveness of residential addiction treatment programs,^4^, ^5^, ^6^, ^7^ these results have further confirmed that residential addiction programs have positive effects on drug abstinence. In particular, the results showed that the use of residential DARCs significantly predicted abstinence from drugs at the individual level in patients with drug use disorders. The residential addictive treatment program may provide each patient with a sense of belonging to a community that shares the same goals of drug abstinence and social recovery, and this may help to keep each patient motivated.^18^ In addition, some previous studies had reported that persons with drug use disorder who were able to abstain from drugs for an extended period showed physiological responses conditioned by drug use, such as withdrawal‐like symptoms and cravings, when they encountered acquaintances who used drugs, places they had used drugs in the past, or objects they had used during past drug use.^19^, ^20^ The use of a residential addictive treatment program might reduce the exposure to stimuli that induce drug reuse. It might be another reason for the results of this study.

Interestingly, the absence of alcohol consumption was associated with the primary outcome, even though the study had excluded those addicted to alcohol. Given the results, the use of nondrinking status as a predictor of relapse and providing appropriate timing for interventions or encouraging patients to engage in the cessation of drinking may be an effective strategy to help patients abstain from drugs. Previous studies have inferred an association between alcohol consumption and relapse of drug use as a characteristic of the patient population.^11^, ^17^, ^21^, ^22^ However, it remains unclear whether drinking predicts drug use. This study is the first to demonstrate that the cessation of drinking significantly predicts individual‐level drug use. A nondrinking situation may reduce the desire to use drugs, as drinking could trigger a recall of the desire to use drugs in patients who would previously drink while using drugs. Furthermore, because the pharmacological effects of alcohol consumption cause cognitive decline,^23^ patients who drink may have difficulty controlling their craving impulses for drug use. In contrast, patients in nondrinking situations may easily control their craving impulses.

In addition, even among those who were not using a residential addiction treatment program, the cessation of drinking was a significant predictor that the patient would not use drugs. This suggests that even patients who leave residential addiction treatment programs and lead independent, healthy social lives (e.g., at home or work) may be able to maintain their continued abstinence from drugs by putting themselves in situations where they do not drink alcohol. Alternatively, as the event of a patient drinking alcohol might be a predictor of relapse of drug use, monitoring the drinking status of patients who have completed a residential addictive treatment program might allow professionals to intervene in a timely manner prior to the patient relapsing into substance use. In addition, among those who did not use residential addiction treatment programs, employment positively influenced the situation in which a patient did not use drugs. The income, relationships, and restored self‐esteem that occurs with employment may positively affect each patient's motivation to abstain from drugs.^24^ For patients with drug use disorders who leave residential addiction treatment programs, continuous employment for more than 6 months is thus another predictor of abstinence, similar to the cessation of drinking.

This study has some limitations. First, the generalizability of the findings is limited, as the data were obtained from DARC patients. These patients are likely to have more severe drug dependence issues than outpatients in hospitals and clinics.^12^, ^13^ However, the findings may be valid in limited settings, such as in similar therapeutic communities. Second, the study did not evaluate the effects of mediating factors. There may have been unknown mediating situational factors between the time‐varying independent variables and nonuse of drugs. For example, as drug treatment recovery programs at other medical institutions are also effective for drug abstinence, patients might be encouraged to undergo combined treatments at such institutions by DARC members during their admission to the DARC. Finally, although nondrinking and drug use status are important variables, there may be reporting bias because of the lack of urine and blood tests. However, as this study aimed to determine whether patients had stopped drinking or using drugs during each follow‐up survey period, urine and blood tests were not used, as they can only assess whether a patient has been drinking or using drugs during the previous week, and we conducted self‐report interviews.

The results suggest that the situation using residential addiction programs can significantly predict no relapse in drug use when compared to the situation not using such programs. The results also suggest that employment and the cessation of drinking are significant predictors of no relapse into drug use, even in situations where the residential addiction program was not used. The results of this study will be helpful for people with drug use disorders seeking social recovery.

## AUTHOR CONTRIBUTIONS

Takuya Shimane designed preliminary experiments. Takuya Shimane and Satoshi Inoura recruited the patients and collected the data. Takuya Shimane established a database of research patients. Satomi Mizuno designed the study, performed statistical analyses, and wrote the manuscript. Takuya Shimane, Satoshi Inoura, and Toshihiko Matsumoto supervised study design, statistical analyses, and manuscript writing. Satomi Mizuno wrote the initial draft of the manuscript. All authors revised and contributed to the final version of the manuscript. All the authors have read and approved the final manuscript for publication.

## CONFLICT OF INTEREST STATEMENT

The authors declare no conflict of interest.

## ETHICS APPROVAL STATEMENT

The study protocol was reviewed and approved by the Ethics Committee of the National Center of Neurology and Psychiatry (NCNP) of Japan (A2016–022). Written informed consent was also obtained from all patients. This study conformed to the provisions of the Declaration of Helsinki.

## PATIENT CONSENT STATEMENT

Patients received an explanation of the purpose of the study, and interested patients could participate after providing written informed consent. Written informed consent was obtained from all patients in the original study, including those in the present study. A copy of the written consent form is available for review upon request.

## CLINICAL TRIAL REGISTRATION

N/A.

## Supporting information

Supporting information.

## Data Availability

The data are unavailable to protect the confidentiality of study patients.
