# Supplementary material for: Situational factors affecting abstinence from drugs: Panel data analysis of patients with drug use disorders in residential drug use treatment
Source: PCN Rep. 2024 Feb 20;3(1):e174. doi: 10.1002/pcn5.174 (PMC11114267; doi:10.1002/pcn5.174)
Supplement: Supplementary file 1 — Supporting information. [file PCN5-3-e174-s001.docx]

**Supporting information**

**Supplementary Table 1. Univariable analysis results**

|  | Crude OR (95% CI) | P-value |
| --- | --- | --- |
| Sex | 1.24 (0.50–3.02) | 0.643 |
|  |  |  |
| Situation of patients’ facility usage  Reference: not using DARC |  |  |
| Not using DARC | Reference | ー |
| Using residential DARC | 4.63 (3.15–6.79) | <0.001 |
| Using outpatient DARC | 2.21 (0.92–5.31) | 0.073 |
|  |  |  |
| Living location |  |  |
| DARC | Reference | ー |
| Home | 0.22 (0.14–0.35) | <0.001 |
| Other facilities apart from DARCs | 0.42 (0.27–0.65) | <0.001 |
|  |  |  |
| Employed situation | 0.82 (0.58–1.18) | 0.293 |
| Situation receiving welfare | 2.41 (1.56–3.72) | <0.001 |
| Situation of no drinking | 4.11 (2.82–6.00) | <0.001 |

Abbreviations: OR, odds ratio; CI, confidence interval.

The reference for patient facility use was based on “not using DARC”.

The reference for living location was based on DARC.

The analysis was adjusted for variables at the follow-up time.

Models (1–5) in Figure 2 were used for this analysis.

**Supplementary Table 2. Univariable analysis results for the stratified analysis of patients not using DARC**

|  | Crude OR (95% CI) | P-value |
| --- | --- | --- |
| Living at home | 0.81 (0.39–1.68) | 0.570 |
| Employment situation | 2.21 (1.09–4.50) | 0.028 |
| Receipt of welfare | 1.23 (0.59–2.59) | 0.580 |
| Non-drinking status | 5.25 (2.99–9.23) | <0.001 |

Abbreviations: OR, odds ratio; CI, confidence interval; DARC, drug addiction rehabilitation center.

The analysis was adjusted for variables at the follow-up time point.

The reference for living location at home was based on the living locations at other facilities apart from DARCs.

Models (i–iv) in Figure 3 were used for this analysis.
